# Introducing the Software CASE (Cluster and Analyze Sound Events) by Comparing Different Clustering Methods and Audio Transformation Techniques Using Animal Vocalizations

**DOI:** 10.3390/ani12162020

**Published:** 2022-08-10

**Authors:** Sebastian Schneider, Kurt Hammerschmidt, Paul Wilhelm Dierkes

**Affiliations:** 1 Bioscience Education and Zoo Biology, Goethe University Frankfurt, 60438 Frankfurt am Main, Germany; 2 Cognitive Ethology Laboratory, German Primate Center, 37077 Göttingen, Germany

**Keywords:** bioacoustics, clustering methods, feature extraction, frequency-modulated vocalizations, multidimensional, vocalization classification, vocal repertoire

## Abstract

**Simple Summary:**

Unsupervised clustering algorithms are widely used in ecology and conservation to classify animal vocalizations, but also offer various advantages in basic research, contributing to the understanding of acoustic communication. Nevertheless, there are still some challenges to overcome. For instance, the quality of the clustering result depends on the audio transformation technique previously used to adjust the audio data. Moreover, it is difficult to verify the reliability of the clustering result. To analyze bioacoustic data using a clustering algorithm, it is, therefore, essential to select a reasonable algorithm from the many existing algorithms and prepare the recorded vocalizations so that the resulting values characterize a vocalization as accurately as possible. Frequency-modulated vocalizations, whose frequencies change over time, pose a particular problem. In this paper, we present the software CASE, which includes various clustering methods and provides an overview of their strengths and weaknesses concerning the classification of bioacoustic data. This software uses a multidimensional feature-extraction method to achieve better clustering results, especially for frequency-modulated vocalizations.

**Abstract:**

Unsupervised clustering algorithms are widely used in ecology and conservation to classify animal sounds, but also offer several advantages in basic bioacoustics research. Consequently, it is important to overcome the existing challenges. A common practice is extracting the acoustic features of vocalizations one-dimensionally, only extracting an average value for a given feature for the entire vocalization. With frequency-modulated vocalizations, whose acoustic features can change over time, this can lead to insufficient characterization. Whether the necessary parameters have been set correctly and the obtained clustering result reliably classifies the vocalizations subsequently often remains unclear. The presented software, CASE, is intended to overcome these challenges. Established and new unsupervised clustering methods (community detection, affinity propagation, HDBSCAN, and fuzzy clustering) are tested in combination with various classifiers (k-nearest neighbor, dynamic time-warping, and cross-correlation) using differently transformed animal vocalizations. These methods are compared with predefined clusters to determine their strengths and weaknesses. In addition, a multidimensional data transformation procedure is presented that better represents the course of multiple acoustic features. The results suggest that, especially with frequency-modulated vocalizations, clustering is more applicable with multidimensional feature extraction compared with one-dimensional feature extraction. The characterization and clustering of vocalizations in multidimensional space offer great potential for future bioacoustic studies. The software CASE includes the developed method of multidimensional feature extraction, as well as all used clustering methods. It allows quickly applying several clustering algorithms to one data set to compare their results and to verify their reliability based on their consistency. Moreover, the software CASE determines the optimal values of most of the necessary parameters automatically. To take advantage of these benefits, the software CASE is provided for free download.

## 1. Introduction

The use of automated evaluations of animal sounds is becoming increasingly common in many areas of ecology and conservation [1]. Animal vocalizations can be used, for example, to monitor populations [2,3] in order to estimate population density [4] or to estimate species diversity [5,6]. Particularly at night or in difficult-to-access and poorly visible areas, such as tropical forests, wetlands, or caves, bioacoustics monitoring is often used [7,8,9,10]. However, even under good conditions where visual observation is possible, bioacoustics methods should be used in addition, since they avoid both observer bias and double counting [11].

In many of these cases, it is helpful, or even necessary, to know the vocal repertoire of the species to be researched. When it comes to conservation, vocalizations can serve as indicators of behavioral states and contexts that provide insight into populations [12]. Information related to reproduction and recruitment, alarm and defense, and social behavior available from monitoring species vocalizations can be translated into potential conservation benefits [12]. Determining the vocal repertoire consequently serves as the basis for many bioacoustics studies. However, the vocal repertoire alone can also provide valuable information about the species in question. It is known for some bird species that a larger vocal repertoire provides a reproductive advantage [13,14]. Furthermore, a correlation between social complexity and the complexity of the vocal repertoire has been demonstrated [15].

For analyzing vocalizations, humans are considered superior to computer-based algorithms in visual pattern recognition, which makes human visual classification an attractive approach [16,17]. However, visual classification also has disadvantages. Visual classification is time-consuming and nearly impossible to implement on large data sets from automatic recording devices. Consequently, the sample sizes used in research may be too small to draw confident conclusions [18]. In addition, visual classification may be affected by subjective errors [19], which makes inter-observer reliability necessary as a measure of the robustness of visual classifications [17]. This subjectivity makes it difficult to compare data sets, in contrast to algorithms, which are particularly suitable due to their continuity. As graphic processing units are becoming more and more efficient, the quality of machine learning and, consequently, the analysis of acoustic signals, is continuously improving [1].

To differentiate the separate vocalizations of an animal and determine the vocal repertoire automatically, clustering algorithms are often used. If the repertoire contains vocalizations that can be used to distinguish individuals of an animal species from one another, it is even possible to survey and monitor populations using such algorithms [20,21]. In addition, clustering algorithms can be used to categorize different animal vocalizations to obtain an overview of the biodiversity in a specific area [22,23]. Clustering algorithms are able to determine differences and/or similarities between single vocalizations to divide them into groups and clusters.

Vocalizations must be available to the clustering algorithms as isolated units. Most commonly, silent gaps between two acoustic elements are used to subdivide units [17]. However, a change in the acoustic features within a vocalization can also provide an occasion for division into two different units if, for example, different meanings are attributed to the units so defined. In the same way, animal sounds repeated in rapid succession can be considered as a whole unit [17]. Particularly in an early stage of a research project, this pre-selection should be conducted manually by a scientist, which means that the entire process cannot be considered automated. However, due to the continuous development of deep learning methods, it is already possible to isolate relevant vocalizations in an automated way, which can enable a fully automated analysis [24]. At this stage, it does not affect whether the vocalization is known or can be assigned. It is merely a matter of recognizing a vocalization as relevant and isolating it from the recording for further processing. The higher the purity of these units and the fewer interfering signals present, the better the algorithm can cluster the units.

There are two types of clustering algorithms: supervised clustering algorithms, which cluster a test data set after being trained with pre-labeled data, and unsupervised clustering algorithms, which can classify an unknown data set without prior labeling and training [17]. In many cases, pre-labeled vocalizations are not available, which makes the application of unsupervised clustering algorithms necessary. Particularly when creating a previously unknown vocal repertoire, unsupervised clustering algorithms are often used [25,26]. However, if different individuals are to be distinguished by possible signature calls, unsupervised clustering algorithms can also be used. There are promising approaches to determining the size of a population by clustering recorded vocalizations that are individually distinguishable [20,21,26].

Algorithms, such as fuzzy c-means clustering or affinity propagation (AP), have proven their efficiency in clustering vocalizations [20,25,26,27]. Nevertheless, there are still a few challenges that make automated clustering in bioacoustics difficult. For example, most clustering algorithms require various parameters to be defined to achieve an optimal result. The values of these parameters that lead to the best result, however, often cannot be determined directly. For fuzzy c-means, for example, a value of “fuzziness” must be chosen, which controls the extent of fuzzy overlapping of the clusters to be formed [25,28]. For AP, however, the parameter “preferences” must be used to determine which vocalizations are most likely to represent a cluster [29].

Besides setting the parameters, it is also crucial how the raw audio signals are transformed before they are clustered using an appropriate algorithm, since, in general, the raw audio files are not used, but the acoustic features of the time domain and frequency spectrum are extracted [17]. The quality of the cluster analysis depends on which and how many acoustic features are extracted [25,30]. Unfortunately, there is no consensus among research groups as to which acoustic features should be extracted, making results difficult to compare. The process of feature extraction typically yields one value per acoustic feature and vocalization. If this acoustic feature changes during the emission of a vocalization, the result is only an average value for this feature. This is not a problem with vocalizations that are constant over time, but can be critical with frequency-modulated vocalizations. In many cases, however, vocalizations are frequency-modulated, such as in cetaceans [31] or birds that live in reverberant habitats, such as forests [17], so that within one vocalization, its acoustic features, such as the fundamental frequency or various frequency bands, can vary over time.

To date, attempts have been made to describe this modulation in frequencies with either a single value, such as a factor of the linear trend of the fundamental frequency [32], or by a vector of an acoustic feature sampled over the signal, such as a frequency contour [33,34]. However, using a single value to characterize a varying frequency response is often not sufficient. Frequency contours better characterize frequency-modulated vocalizations and can be compared using dynamic time warping (DTW), for example [33,34]. However, only a single acoustic feature is used, which means that, in some cases, classification by a variety of point-measured acoustic features gives better results [34]. A solution where a variety of acoustic features are sampled over the signal for high-resolution characterization would combine many advantages and thus increase the quality of classification.

Another major challenge in clustering vocalizations is to assess the reliability of the results. Silhouette coefficients are often used in this regard [20,34]. This may work well if several iterations of a clustering method are conducted under the same conditions, for example, to set necessary parameters as optimally as possible. Silhouette coefficients, however, seem rather unsuitable for evaluating a clustering result in terms of its reliability, as the silhouette values become worse with an increasing number of features [25]. Accordingly, it is difficult to evaluate the clustering result obtained in terms of its quality. In summary, four major challenges arise in the unsupervised clustering of vocalizations: (1) extraction of a large number of meaningful acoustic features, which also represent the frequency modularity over time; (2) choosing a suitable clustering method; (3) the optimal setting of necessary parameters; and (4) verifying the reliability of the clustering result.

To overcome these challenges, the software CASE was developed as part of this work (the link to the free download is provided in Appendix A).

To be able to better cluster frequency-modulated vocalizations, we used an approach of feature extraction, where the acoustic features are extracted in a windowed manner. In this way, one value per window and acoustic feature is obtained, and the resulting multidimensional feature matrix is used as the input for the clustering method, whereby the values of the acoustic features changing along a vocalization are better represented and should lead to an increase in classification quality. This alternative procedure is compared with a common procedure in which each acoustic feature is represented by one average value. In addition, it is tested whether certain procedures and algorithms also yield good cluster solutions if no acoustic features are extracted, but the audio signals are merely transferred into the frequency domain using a Fourier transformation, so the spectra are used as inputs for clustering methods. Consequently, the result would be independent of the number and selection of acoustic features, which would make a quantitative comparison of the determined repertoires possible. Using spectrograms in addition has the advantage that the audio signals are windowed, which means that changes in frequencies over time are taken into account, and thus frequency-modulated vocalizations are better represented.

The clustering methods used by the software CASE have been modified so that the optimum values for most parameters are determined in an automated process. Thus, after an iterative setting of different values for the corresponding parameter, the value that yields the most stable cluster solution is taken.

Both established methods and methods that have not yet been used in bioacoustics, but have proven themselves in other areas, were applied. All clustering algorithms used by the software CASE have the advantage that they do not require the exact number of clusters or the initial starting points a priori. The established clustering algorithms include fuzzy clustering [25,27] and AP [20,26]. Fuzzy clustering has the advantage that the data points are not sharply delimited from each other, but each data point is assigned a certain degree of membership to a cluster. This makes it possible to better cluster vocalizations that have smooth transitions instead of distinct boundaries, as is the case with graded vocalizations, for example. However, the fuzzy clustering algorithm uses several parameters that need to be estimated, such as the maximum number of clusters to be determined, as well as the degree of “fuzziness”. Thus, the quality of the result depends on the selection of values for the parameters [25]. For AP, parameters that influence the resulting number of clusters must also be set in advance [29]. However, the correct selection of the corresponding values is not evident. AP is often particularly used in combination with DTW [20,26]. DTW compares two signals with each other and indicates the Euclidean distance between the two—in simple terms, how much they differ. The advantage is that the signals can be of different lengths, and DTW finds the optimal match by stretching the signals. However, this has the disadvantage that vocal types, which differ mainly in duration, are difficult to distinguish. With DTW the signals to be compared can be provided as a matrix, making it possible to directly compare two spectrograms or the data matrices of the windowed feature extraction. This is also possible via cross-correlation, allowing for the classification of windowed acoustic features as well. Cross-correlation (Xcorr) was first used in 1987 by Clark et al. to compare spectrograms [35], and has since often been used in bioacoustics. Due to the stepwise comparison of the single data points with each other, Xcorr is slower than the other classifiers presented here, especially for matrices. The k-nearest neighbor (kNN) classifier is also used to classify species by their vocalizations [36,37], but is not able to compare two matrices, which requires a further transformation of the windowed data (see Materials and Methods). Nevertheless, kNN is one of the oldest, simplest, and most accurate algorithms for pattern classification and is, therefore, widely used [38]. Community detection (CD), on the other hand, is a clustering algorithm that has not yet been used in bioacoustics, but is often used in clustering networks, such as social networks, or even in neurobiology [39,40]. The strengths of CD lie primarily in its high speed and diverse application possibilities with high-dimensional data [41,42]. In addition, a new hierarchical density-based clustering algorithm (HDBSCAN), introduced by Campello et al. in 2013 [43], will be applied and compared, which was first used in bioacoustics in 2020 [44,45]. This algorithm has the advantage that outliers are recognized as noise and discarded, so data points that are difficult to assign are ignored and do not negatively affect the clustering result.

**Figure 1:**
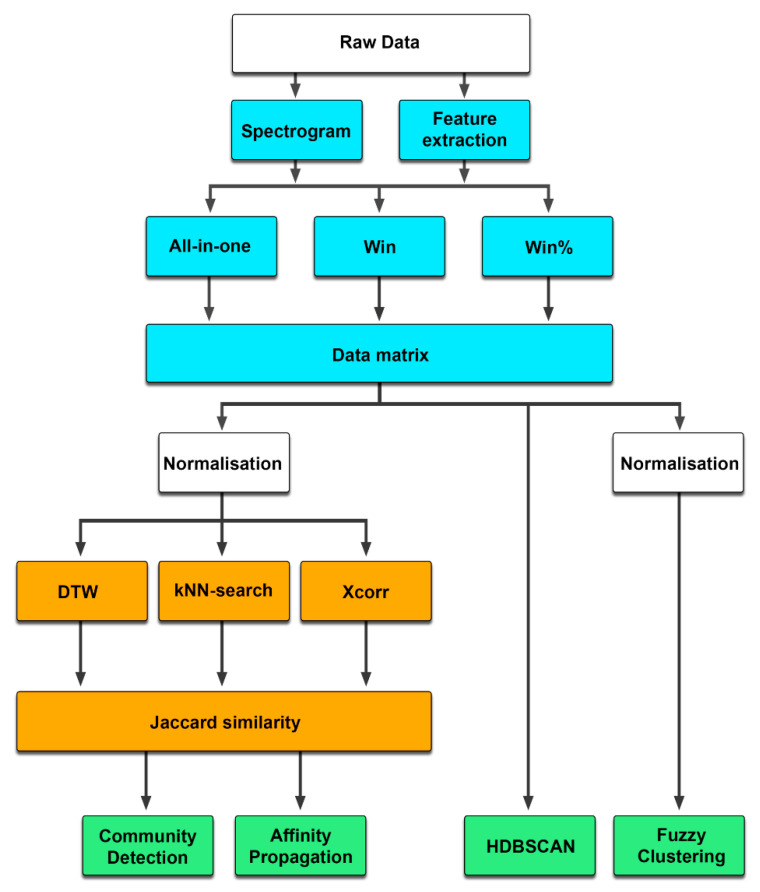
Sequence of clustering procedures. Possible combinations are shown, resulting in 48 different procedures.

Accordingly, four different clustering algorithms (CD, AP, fuzzy clustering, and HDBSCAN) were applied and compared in this paper, of which CD and AP relied on prior classification and were combined with all three classifiers (DTW, kNN, and Xcorr) to show possible differences. Additionally, the audio signals of the vocalizations were transformed in different ways before being used as inputs—once according to the usual procedure, where each acoustic feature was represented by one value (All-in-one), and also in windowed form (win and win (%)), where each acoustic feature was determined per window (Figure 1).

**Figure 2:**
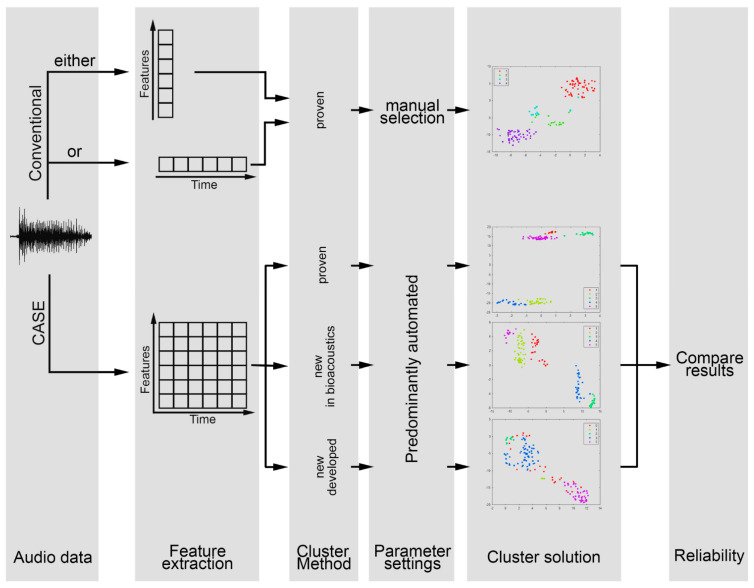
Illustration of the advantages of the software CASE in comparison with conventional approaches using one-dimensional feature vectors as inputs for a single clustering algorithm.

Due to the variety of clustering methods, it is possible to compare the results of different algorithms. In this way, a stable and reliable clustering solution can be obtained through similar results from different methods. For this purpose, the software CASE offers a procedure to compare the determined labels and evaluate their concordance (see Section 2.4. Verifying Cluster Quality). Figure 2 further illustrates the advantages of the software CASE presented here and how it overcomes previous challenges compared with conventional approaches.

We used two different data sets here to test the clustering methods. On the one hand, vocalizations of two harpy eagles (*Harpia harpyja*) at Nuremberg Zoo were used, and cohesion calls of a group of giant otters (*Pteronura brasiliensis*) at Dortmund Zoo were used on the other hand. So far, no work has been published about the vocal repertoire of the harpy eagle. Using the harpy eagle vocalizations, how well the clustering methods can distinguish between different slightly frequency-modulated vocalizations of the same species will be tested. For the giant otters, however, only one vocalization was used: the so-called cohesion call. The cohesion calls of giant otters are known to signal the identity of the individual [46], i.e., the individuals can be distinguished by this vocalization. Therefore, a data set with these vocalizations was used to determine if the clustering methods could distinguish the individuals using this highly frequency-modulated vocalization and thus determine the number of individuals. Clustering methods that perform this task could be used, for example, to survey populations in poorly visible terrain, in the ocean, or murky waters.

So far, there have only been a few studies comparing a variety of clustering methods based on animal vocalizations to discuss their usefulness for different challenges. Often only two to three algorithms are compared, or the algorithms are used for very specific questions [20,33]. In this study, 48 different procedures were applied to two different problems: the clustering of different vocalizations of one species to create a repertoire, and the clustering of specific vocalizations with individual differences to determine the number of individuals.

The method presented here, which uses windowed sampling of acoustic features, provides the mapping of acoustic features in multidimensional spaces required by recent studies to quantify the variation between acoustic signals prior to comparative analysis [34]. The insights gained here should show that a windowed extraction of various acoustic features can improve the quality of a clustering result. Future studies can benefit from the comparison of different approaches and clustering methods by showing where the strengths and weaknesses of the different algorithms lie. Thus, depending on the existing conditions, which transformation of the recorded data in combination with which clustering algorithm is most sensible can be determined. The goal is to introduce an approach by which the acoustic features of vocalizations can be mapped multidimensionally to achieve clustering results that are as accurate and reliable as possible.

## 2. Materials and Methods

### 2.1. Data Collection and Study Site

**Figure 3:**
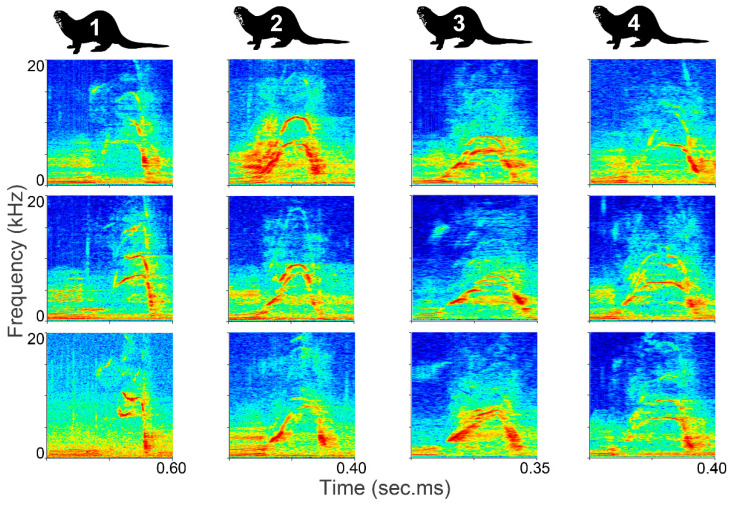
Cohesion calls of the giant otters. Three of the selected cohesion calls per individual are shown.

This study adhered to the EAZA Code of Ethics and advice on the husbandry of zoo and wildlife species in captivity. Further ethical approval was not required for the animal study because only observational data were collected, with no change whatsoever to the animals’ environment or management. Consent was obtained from the owners for the participation of their animals in this study. Vocalizations of harpy eagles and giant otters were used to test the clustering methods. For the giant otters, only cohesion calls were used [46]. These were recorded at Dortmund Zoo in Germany during May 2018. Eight “the t.bone” EM 9900 directional microphones (Thomann, Burgebrach, Germany) were positioned around the outdoor enclosure, covering most of the areas within. The recordings were produced using two linked Tascam DR-680 MK2 multitrack field recorders (TEAC, Tama, Tokyo, Japan) and a sample rate of 96 kHz. At the time of recording, there were four adult individuals in the enclosure. Thirty cohesion calls were selected, which were free of interfering sounds, had a good signal-to-noise ratio (SNR), and could be assigned to the corresponding individual (Figure 3). Five, six, seven, and twelve vocalizations could be assigned per individual.

**Figure 4:**
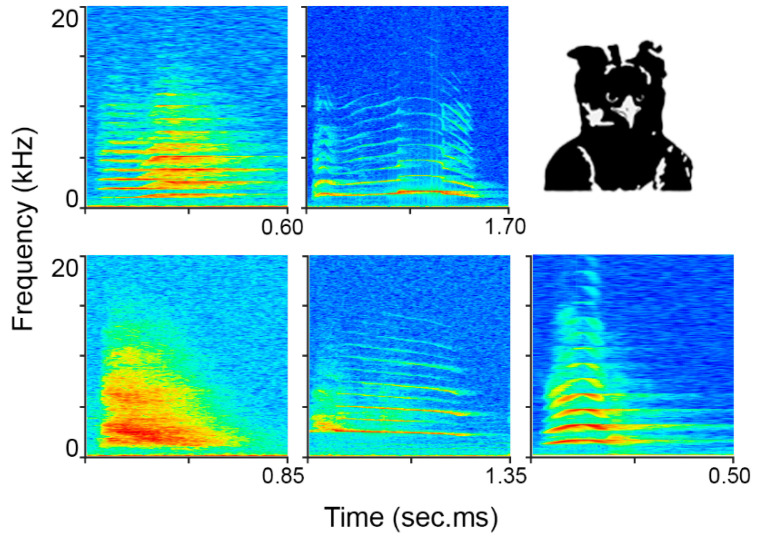
The five different vocal types of the harpy eagles that were used.

The harpy eagle vocalizations were recorded from a breeding couple in September 2019 at Nuremberg Zoo. Both individuals were kept in separate, but adjacent, aviaries. A Sennheiser ME 66 directional microphone (Sennheiser, Wedemark-Wennebostel, Germany) and a Tascam DR 100 MK2 (TEAC, Tama, Tokyo, Japan) recorder were used. The recordings were produced with a sample rate of 48 kHz. Only vocalizations were selected that were free of interfering sounds and that showed a good SNR. In addition, units that occurred fewer than 5 times were discarded. This resulted in 141 vocalizations being used (Figure 4).

For the classification of the harpy eagle and giant otter vocalizations, unsupervised clustering methods were used. The clustering analysis, as well as data transformation, was performed using MathWorks MATLAB 2020a (The MathWorks Inc., Natick, MA, USA).

### 2.2. Data Transformation

**Figure 5:**
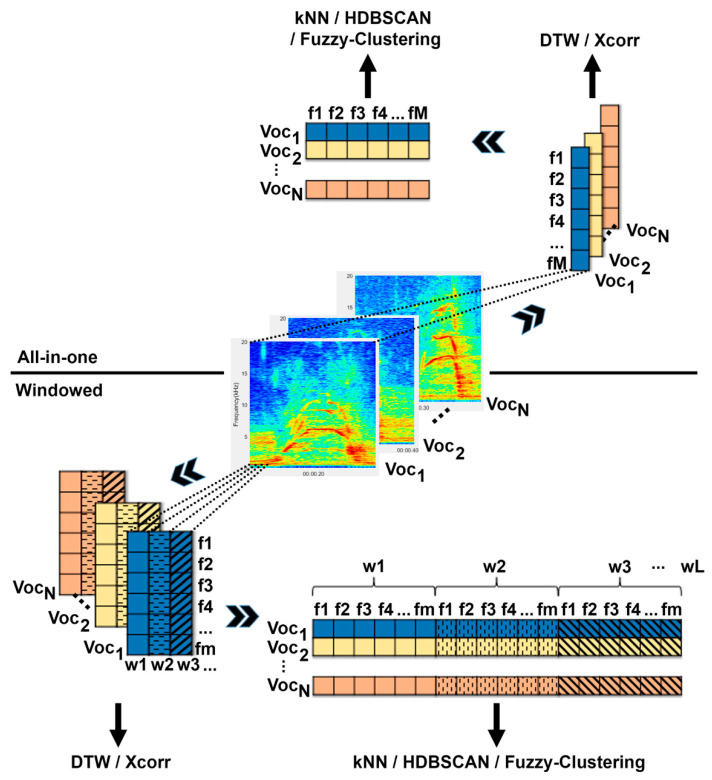
Process of data transformation using the All-in-one method (**upper half**) and the windowed method (**lower half**). All-in-one: For each vocalization (Voc1 to VocN) M acoustic features are extracted or M frequency domain representations are determined using FFT. These can then either be classified directly via DTW or Xcorr, or are transferred to an NxM matrix for further processing via kNN, HDBSCAN, or fuzzy clustering. Windowed: Each vocalization (Voc1 to VocN) is divided into L windows (w1 to wL) from which m acoustic features are extracted or m frequency domain representations are determined by FFT. These mxL matrices can then either be classified directly via DTW or Xcorr, or are transferred into an NxM matrix (where M = L × m) for further processing via kNN, HDBSCAN, or fuzzy clustering.

We compared different types of data transformation that can be used for clustering vocalizations. As a basis for further processing, the volume of vocalizations was normalized to 1. The recorded audio files were filtered using a fifth-order highpass Butterworth filter at 600 Hz for the harpy eagle vocalizations and at 150 Hz for the giant otter vocalizations. All vocalizations then were either transferred to the frequency domain, or the acoustic features of the audio files were extracted. Depending on whether the All-in-one approach was used or each vocalization was windowed, the data transformation procedure differed (Figure 5).

#### 2.2.1. All-in-One

The “All-in-one” approach is the term we use to describe the conventional procedure for feature extraction in bioacoustics. For each acoustic feature, one value is determined, which is representative of the entire vocalization. Some of these acoustic features are collected in a windowed manner, but are combined into one value using their median. This is not a problem for consistent vocalizations, but becomes inaccurate as soon as the vocalization is frequency-modulated, i.e., the values of acoustic features change along a vocalization. To transfer a vocalization into the frequency domain, a fast Fourier transformation (FFT) is performed for the entire vocalization. The resulting spectra (one spectrum for each vocalization) are combined in an N × M matrix, where N is the number of vocalizations and M is the frequency spectrum. The amplitude of the frequencies is determined from 0 to 20 kHz every 10 Hz, resulting in M = 2000. When extracting acoustic features, an average value for each feature is determined for a vocalization. This results in an NxM matrix, where N is the number of vocalizations and M is the number of acoustic features. For frequency domain representation, the spectrum is calculated using FFT. For more details about the acoustic features, see Section 2.2.3. Feature extraction.

#### 2.2.2. Windowed

To determine the chronological sequence of the characteristics of a vocalization, each vocalization was windowed and the acoustic features or the frequency spectrum for each window were determined. Since the vocalizations were of different lengths, two possible methods were compared. Firstly (win), the windows were all the same size, with each window having a length of 2048 samples and overlapping 50% with the neighboring windows. If it is necessary for the remaining procedures that all vocalizations have the same length (kNN, HDBSCAN, and fuzzy clustering), the missing windows that result from the difference in the longest vocalization are filled with zeros. Secondly (win%), the window size is adjusted to the number of windows of the shortest vocalization, so that the shortest vocalization has a window length of 2048 samples, and all other vocalizations have as many windows, but of different sizes. Again, the windows overlap by 50%. If the vocalizations are to be transformed into the frequency domain, the spectrogram is determined for each vocalization using a short-term Fourier transformation and a Hamming window. The frequency range includes 0 to 20 kHz, with a step size of 20 Hz, which results in a matrix with 2000 rows, with each column containing the corresponding power spectrum of a window. If feature extraction is performed instead, all acoustic features are extracted separately for each window. For more details about the acoustic features, see Section 2.2.3. Feature extraction.

Since windowing creates a separate matrix for each vocalization, but some clustering methods require a single matrix as an input, where the information of a vocalization is arranged in a single row, the windowed matrices have to be rearranged for the kNN, HDBSCAN, and fuzzy clustering methods. For this purpose, each matrix is reshaped into a vector, with the individual windows being positioned behind each other. By merging the vocalization vectors, the desired N × M matrix is created, where N corresponds to the number of vocalizations and M to the number of acoustic features multiplied by the number of windows per vocalization (Figure 5).

#### 2.2.3. Feature Extraction

For feature extraction, acoustic parameters (features) are calculated mathematically. Since the clustering algorithms always require the same dimensionality as an input, the same acoustic features must be used for all vocalizations in a data set. Twenty-seven acoustic features were extracted for each vocalization or each window of a vocalization. As in windowed vocalizations, the number of windows reflects the duration—this feature was omitted in this case, resulting in 26 acoustic features. With the method Win%, however, the duration of the entire vocalization is given in each window as an acoustic feature, since each vocalization is divided into the same number of windows. For the windowed extraction of acoustic features using the spectrum, features are determined using the spectrum of each time window. For the “All-in-one” method, all features were estimated using the spectrum of the whole vocalization. Table 1 describes each acoustic feature.

### 2.3. Clustering Methods

The matrices obtained through the data transformation described above were used as inputs for the clustering methods. Different clustering algorithms were used for comparison. For better comparability of the algorithms, the seed of the random number generator was fixed so that randomly chosen numbers were always selected identically for all algorithms. This can be useful, for example, if vocalizations are randomly selected as initial cluster centroids. Since some of the clustering algorithms require a similarity matrix, different methods for creating these similarity matrices were compared. In the following, the clustering algorithms, as well as the corresponding procedures for creating the similarity matrices, are described.

#### 2.3.1. Similarity Matrices

To describe how similar the different vocalizations within the data matrix are, various classifiers were chosen and compared. Before any of the following methods were applied, the data matrices were normalized by zscore. The similarity matrix was calculated in two steps. First, the distance between the vocalizations was determined by DTW or kNN, or the concordance was determined by Xcorr. The result was an N × N matrix, where N is the number of vocalizations. Each row corresponds to one vocalization and the columns contain the index number of the vocalization, which best matches the vocalization of the corresponding row, in descending order. Accordingly, each row contains, in the first column, the index number of the vocalization that fits best, and, in the last column, the one with the worst fit. For the second step, only the k best matches were used, and with the resulting N × k matrix, the Jaccard similarity was determined. Jaccard similarity is defined as:

(1) $$\mathrm{jaccard}\;(A,B)=\frac{\left|A\cap B\right|}{\left|A\cup B\right|}$$

where *A* is the ith row of the N × k matrix and *B* is the jth row of the N × k matrix. The value for k that should be used is calculated automatically by making multiple different assumptions for k and, accordingly, all resulting similarity matrices are applied to the selected clustering algorithm. For each clustering result, the determined number of clusters was noted and the corresponding average of the silhouette values *Si* was calculated.

(2) $$Si=\frac{\left(b-a\right)}{\max(a,b)}$$

where *a* is the average distance from the nth vocalization to the other vocalizations in the same cluster as n, and *b* is the minimum average distance from the nth vocalization to the vocalizations in a different cluster, minimized over clusters. 

The similarity matrix for which k represents the most frequently occurring number of clusters and the *a* silhouette mean value is as large as possible is then used for clustering using the corresponding algorithm.

#### 2.3.2. Dynamic Time Warping

DTW [48,49] refers to an algorithm that compares two signals with each other. For this purpose, the Euclidean distance between two signals is calculated, and each element (for matrices each column) of the two signals is repeated as long as necessary until the Euclidean distance is smallest. The result is an N × N matrix with the smallest possible distances between the vocalizations. From this N × N distance matrix, the indices of the k smallest distances are transferred in ascending order into a new N × k matrix, where the index with the smallest distance is at the beginning, and the one with the kth largest distance is at the kth position.

#### 2.3.3. k-Nearest Neighbor Search

KNN search [50] determines, for each object N_i_ (in this case, vocalizations), its k nearest neighbors with the smallest distance. As an input, an N × M matrix is used, where N is the number of vocalizations and M is the number of acoustic features (Figure 5). To calculate the distance between the objects, the Euclidean distance was used here. The best value for k was determined automatically as described above using the best silhouette value of the most stable number of clusters. The result is an N × k matrix with the k nearest neighbors for each object N_i_, which is further used to calculate a corresponding similarity matrix using the Jaccard similarity.

#### 2.3.4. Cross-Correlation

Xcorr determines the similarity between one signal and the shifted copies of a second signal. A coefficient is obtained for each shift. The greater the match, the higher the value. It can be used for one-dimensional vectors (as they are provided using the All-in-one approach), but also for two-dimensional matrices (as they are provided when vocalizations are windowed). For an M × N matrix X and a P × Q matrix H, the cross-correlation coefficients C are calculated by:

(3) $$\begin{array}{ll} C(k,l)= & \sum_{m=0}^{M-1}\sum_{n=0}^{N-1}X(m,n)\bar{H}(m-k,n-l)\\ & -(P-1)\leq k\leq M-1\\ & -(Q-1)\leq l\leq N-1 \end{array}$$

for two vectors *x_n_* and *y_n_* with N samples, the cross-correlation coefficients *R* are calculated by:

(4) $$R_{xy}(m)=\left\{ \begin{array}{ll} \sum_{n=0}^{N-m-1}x_{n+m}{\bar{y}}_{n},\; & m\geq 0\\ {\bar{R}}_{yx}(-m),\; & m\geq 0 \end{array}\right.$$

where *k* and *m* are the row indices and l and n are the column indices. The bard over *H*, *y*, and *R* denote complex conjugation. If vectors are used (All-in-one) that do not have the same length, the vector with fewer elements is brought to length *N* by adding zeros at the end. Using matrices (Windowed), the number of columns can differ and no zeros are added.

Xcorr is the basic statistical approach to image registration [51]. Here, cross-correlation was used similarly if the spectrograms or spectrums of the vocalizations served as inputs. For each cross-correlation of two vocalizations, the largest cross-correlation coefficient was entered into a matrix as the value for the match, resulting in an N × N matrix where each row and column correspond to one vocalization, and each cell contains the maximum correlation coefficient of the corresponding vocalizations. For each row, the indices of the columns with the k-largest correlation coefficients are transformed into a new N × k matrix, and a corresponding similarity matrix is calculated using the Jaccard similarity.

#### 2.3.5. Community Detection

One of the most relevant features of graphs representing real systems is community structure, or clustering, i.e., the organization of vertices in clusters, with many edges joining vertices of the same cluster and comparatively few edges joining vertices of different clusters [39]. Here, an agglomerative hierarchical clustering method was used, which was published by M. E. J. Newman in 2004 [41]. This algorithm initially assumes a state in which each vertex alone forms one of *N* communities, i.e., clusters, and then joins more and more communities together in pairs, which can be seen as a hierarchical dendrogram. For each step, a value for the quality of community formation is determined, i.e., the modularity *Q*. *Q* subtracts the expected fractions of edges in a community from the observed fractions of edges in the same community:

(5) $$Q=\sum_{i=1}^{N}(e_{ii}-a_{i}{}^{2})$$

where *N* is the number of communities, *e_ii_* is the fraction of edges in community *i*, and *a_i_* is the fraction of all ends of edges that are attached to vertices in community *i* [41]. The step with the highest value for *Q* defines the best cluster solution and, therefore, the number of communities.

An adjacency matrix was used as an input, in which the edges between the vertices were noted. Since, in our case, a Jaccard similarity matrix was used, the edges between the vertices were weighted.

#### 2.3.6. Affinity Propagation

AP clusters data by determining representative members from the data points for each cluster, the so-called “exemplars “. Real-valued messages are exchanged between the data points to decide whether a data point becomes an exemplar or to which exemplar it is assigned. The messages are exchanged until a high-quality set of exemplars, and thus the best number of clusters, gradually emerges [29]. This process is influenced by the similarity matrix, which serves as an input, and the “preferences”, a previously defined parameter, containing one value per data point (in this case, vocalizations) that influences whether a data point is chosen as an exemplar and how many exemplars are determined. Thus, the preferences influence the number of clusters significantly. The lower the values set for the preferences, the fewer clusters generated.

Originally, AP was published by Frey and Dueck in 2007 [29]. Here, however, we used the adaptive AP by Kaijun Wang et al., who further developed the algorithm [52]. The adaptive AP method was proposed to overcome the limitations of AP, including the adaptive scanning of preferences to search the space of the number of clusters for finding the optimal cluster solution, adaptive adjustment of damping factors to eliminate oscillations, and adaptive escaping from oscillations when the damping adjustment technique fails [52]. To calculate the preferences, the median of the input similarities is first determined [29], which results in different values for the vocalizations. To determine the number of clusters as reliably as possible, an additional procedure was applied, which uniformly reduced the values for the preferences and determined the corresponding number of clusters until a minimum of clusters (in this case, 3) was obtained. Starting from these “start-preferences”, the preferences increased step-by-step up to a maximum value of 1 (since the Jaccard similarity can be a maximum of 1). For each step, both the silhouette value and the number of clusters are noted to define the most stable cluster solution at the end of the run. 

#### 2.3.7. HDBSCAN

HDBSCAN is a divisive hierarchical clustering method that forms clusters according to the density of data points and discards unassignable data points as noise. Two parameters must be defined in advance: the number of nearest adjacent data points over which the core distance is determined (minpts), and the minimum number of data points required to form a cluster (minclustsize).

To find areas with a high density of data points, a core distance is calculated for each data point by calculating the distance to the minpts nearest neighbor. Using these core distances, a minimum spanning tree is built and then converted into the hierarchy of connected components. Using the parameter minclustsize, the cluster hierarchy can be condensed. This is achieved by discarding any new cluster that contains fewer than minclustsize data points at each division. How long a cluster remains is an indicator of its stability, based on which the number of clusters is determined. All data points that have been discarded and are therefore not assigned to a cluster are declared as “noise” [43,53].

In this paper, the parameters minpts and minclustsize were both assigned a value of 2, since, in some cases, higher values did not lead to any clusters.

#### 2.3.8. Fuzzy Clustering

In the fuzzy set theory [54,55], data points are not sharply delimited from each other, but each data point is assigned a certain degree of membership to a cluster. The membership can take values between 0 and 1. The greater this value is, the stronger the affiliation to the corresponding cluster. Thus, the determination of centroids is less affected by data points that do not contain a high membership value and can be considered as outliers. Here, we use a fuzzy c-means clustering algorithm, which requires a maximum number of clusters and the “fuzziness” as an input parameter, based on which the most stable cluster solution is determined [25,28]. The fuzziness parameter (µ) can be used to control the amount of fuzzy overlap of clusters. The higher the value, the greater the fuzzy overlap. The closer the value is to 1, the crisper the boundaries with other clusters. Since a value of 1 would give the same results as k-means, a value greater than 1 must be specified. 

By iteratively increasing µ, centroids of similar clusters approach each other. If the Euclidean distance of two centroids falls below a certain threshold (here ɛ = 0.01), the corresponding clusters are merged, whereby the number of clusters is decimated step-by-step. The number of clusters that persists over most values for µ is considered as the best cluster solution. Based on the paper by Wadewitz et al., a relatively large maximum number of 15 clusters was determined, and the fuzziness ranged from 1.1 to 2.5, increasing by 0.05 every iteration [25].

### 2.4. Verifying Cluster Quality

To verify the quality of the clustering procedures, the vocalizations of both harpy eagles and giant otters were classified and labeled a priori. The giant otter vocalizations were linked to the corresponding individual emitting the vocalization via sound localization using the software LASER [56]. The exact experimental setup and method can be found in the publication on the software LASER [52]. Only vocalizations were used where the individual emitting the sound could be identified clearly, which made human rating unnecessary. 

According to the author’s knowledge, the vocalization repertoire of the harpy eagle has not yet been defined, so a classification of the vocalizations was carried out by the author. Five vocal types were selected, which were separated by silence and could be distinguished both visually by their spectrograms and audibly (Figure 4). 

In addition, the selected vocalizations of harpy eagles and giant otters were labeled by five inexperienced observers to compare the labeling of the clustering procedures with human ones. These five observers classified the vocalizations visually via a spectrogram and audibly. The observers had no information about the number of animals or the number of different vocal types that could be found. To determine the rater reliability, the labels of the raters were compared with each other using Normalized mutual information (NMI). For the harpy eagle vocalizations, the average NMI value was 0.87 (standard deviation = 0.05), and for the giant otters, the average NMI value was 0.3 (standard deviation = 0.12).

NMI was used to verify the concordance of the labeled data. NMI is defined as:

(6) $$NMI\left(x,y\right)=\frac{I\left(x,y\right)}{\sqrt{H(x)*H(y)}}$$

where *I(x, y)* is the mutual information between *x* (a priori label) and *y* (label to be checked), and *H(x)* and *H(y)* are the entropies of *x* and *y,* respectively. NMI takes values between 1 and 0, where 1 corresponds to a perfect match and values close to 0 represent a random distribution. 

When comparing the labels determined as a result of HDBSCAN with the a priori labels, all vocalizations labeled as 0 were discarded as they corresponded to “noise”. It should be noted that, while a high NMI value indicates a high degree of consistency in labeling, it does not indicate whether the correct number of clusters has been found. Thus, the number of clusters determined was subtracted from the actual number to determine the deviation. The absolute value of this difference is called Δ-Cluster. To determine which of the tested methods provided suitable results, threshold values were set for NMI and Δ-Cluster. For the NMI value, we followed Kershenbaum et al. (2013) [33], in which a selection of clustering algorithms achieved an NMI value between 0.52 and 0.79. For this reason, the NMI threshold value was set to 0.5. Since the number of clusters should be 4 for giant otters and 5 for harpy eagle vocal types, a maximum deviation of one cluster was tolerated for Δ-Cluster (Δ-Cluster ≤ 1). For larger numbers of clusters, a larger value for Δ-Cluster would be tolerable.

## 3. Results

**Figure 6:**
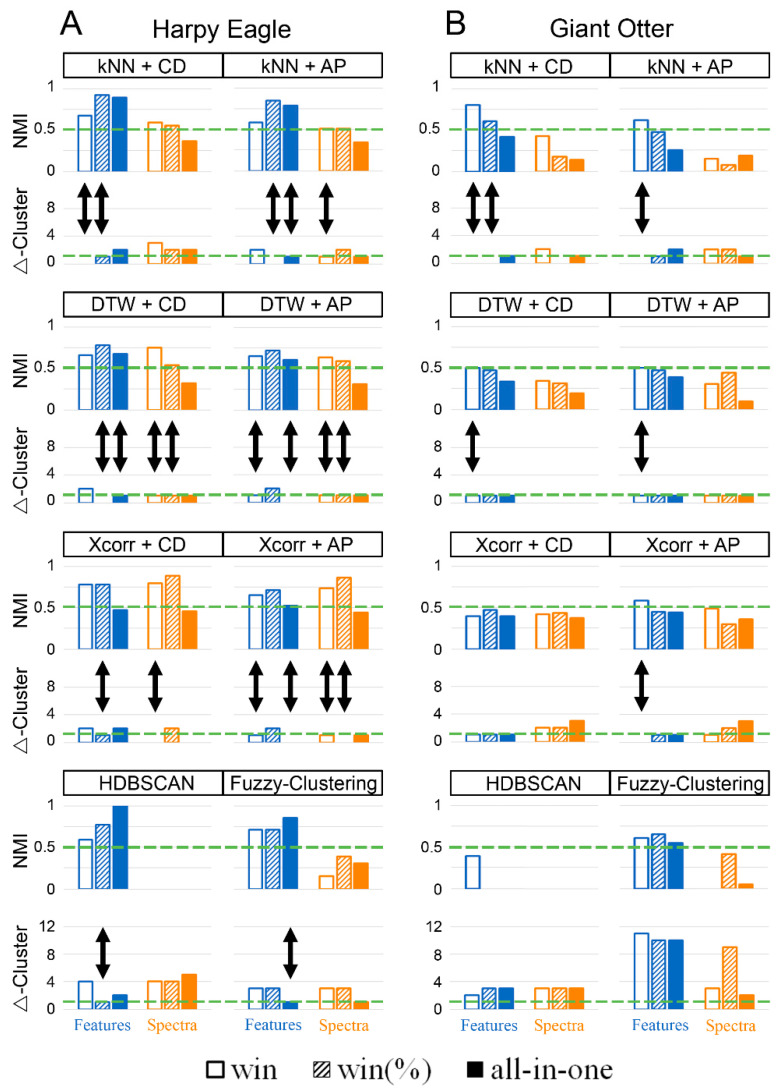
Results of the clustering methods obtained using the harpy eagle vocalizations (**A**) and the cohesion calls of the giant otters (**B**). The green dashed lines mark the threshold value that must be exceeded for NMI values (>0.5) and fallen below for Δ-Cluster values (≤1). The double arrows mark the cluster solutions that fulfill both the threshold value for NMI and the threshold value for Δ-Cluster.

To evaluate and compare the quality of the different clustering algorithms, the match between the result of each clustering procedure and the a priori labels was determined via NMI. Since the resulting value for NMI only reflected the match of the labeling, and not whether the correct number of clusters was calculated, the deviation from the actual number of clusters (Δ-Cluster) was additionally included as a quality criterion. The results are shown in Figure 6.

As expected, clustering the different vocal types of the harpy eagles worked better than clustering the individually different cohesion calls of giant otters (Figure 6; Table 2 and Table 3). After all, five of the tested procedures (kNN + CD, kNN + AP, DTW + CD, DTW + AP, and Xcorr + AP) could distinguish the cohesion calls of the giant otters reliably (double arrows Figure 6B; Table 2). Furthermore, the clustering methods could comply with the set threshold values only if acoustic features were extracted in a windowed manner. The NMI values for these three methods ranged between 0.50 (DTW + CD and DTW + AP) and 0.8 (kNN + CD). Neither the spectra nor the All-in-one data preparation produced sufficiently good results when clustering the giant otter vocalizations.

When clustering harpy eagle vocalizations, none of the algorithms particularly stood out if acoustic features were extracted for the corresponding method (Figure 6A; Table 3). Examining the procedures that complied with the threshold values for NMI and Δ-Cluster, it is noticeable that, except for fuzzy clustering, the NMI of at least one of the windowed data was always higher than the NMI that would result if the corresponding algorithm used All-in-one-prepared data. For windowed acoustic features, the NMI ranged between 0.65 (DTW + AP) and 0.92 (kNN + CD), and between 0.53 (Xcorr + AP) and 0.85 (fuzzy clustering) for acoustic features extracted using the All-in-one procedure. When spectra were used as input, only DTW or Xcorr were capable of producing reliable results that complied with the threshold values. AP, in combination with kNN, also complied with the set threshold values, but only very narrowly, with an NMI of 0.51, and only if vocalizations were windowed. None of the methods were able to achieve sufficiently good results using spectra in combination with All-in-one-prepared data. When the vocalizations were windowed, the NMI values of the methods complying with the threshold ranged between 0.51 (kNN + AP) and 0.87 (Xcorr + AP).

Since one of the major strengths of the software CASE is the quick comparison of different clustering results, the labels of the reasonably selected clustering methods were compared using NMI. In this way, whether the results obtained were reliable could be assessed, even if the membership of the individual vocalizations was not known in advance. Table 4 and Table 5 show how similar the labels of the clustering procedures are to one another. The higher the NMI value, the greater the concordance of the determined cluster solutions.

For the vocalizations of the harpy eagles, the NMI values were frequently above the defined threshold of 0.5, and exceeded a value of 0.9 in some cases (Table 4). The concordances of the assigned labels were correspondingly large, indicating reliable clustering results for many clustering methods. The median, as well as the most frequent number of clusters determined, was 5, which corresponded to the number of clusters determined a priori.

For the more difficult-to-distinguish giant otter vocalizations, only a few clustering methods still exceeded an NMI value of 0.5 with a simultaneously low deviation in the number of clusters (Table 5). However, it was precisely the methods that showed the greatest consistency (knn + CD, knn + AP, DTW + CD, and DTW + AP) that also corresponded best to the a priori labels. The median corresponded to 3.5, which was close to the four clusters identified a priori. A reliable result could be determined accordingly via the comparison of the labels, even if the number of clusters and the assignment of the vocalizations were not known.

To evaluate how well the algorithms performed compared with human subjects, five inexperienced observers visually classified the vocalizations. For the harpy eagles, the NMI values ranged from 0.85 to 0.95, with a maximum Δ-Cluster of 1, whereas, for the giant otters, none of the observers were able to classify the cohesion calls sufficiently well according to the threshold values for NMI and Δ-Clusters. The values for NMI here ranged between 0.3 and 0.51, with a maximum Δ-Cluster of 3 (Table 6).

## 4. Discussion

As expected, the vocal types of the harpy eagles could be clustered better than the cohesion calls of the giant otters. Nevertheless, five of the automated methods (kNN + CD, kNN + AP, DTW + CD, DTW + AP, and Xcorr + AP) succeeded in clustering the cohesion calls of the giant otters better than the human subjects (Table 4). Additionally, the vocalizations of the harpy eagles were classified only slightly better by human visual clustering than by certain clustering methods (Table 4). The NMI achieved by kNN + CD and win% transformed vocalizations was 0.9231, and thus nearly equal to the mean value of the NMI values achieved by human visual clustering (0.9166). This suggests that the automated classification of vocalizations is not only much faster, but also provides a result of similar high quality. 

The clustering of the giant otters’ cohesion calls was only possible via extracted acoustic features, but not via spectra. In general, it can be said that the clustering of spectra only seems to make sense using DTW or Xcorr. However, if a similarity matrix is being created with one of these two methods, robust results can be achieved by community detection, as well as by affinity propagation. The results obtained by Xcorr are usually a little bit better than those obtained by DTW when using a spectrogram. However, the determination of the similarity matrix by Xcorr takes much more time. In our case, DTW took about 1 minute (win) or 30 s (win%), whereas Xcorr took 60 minutes (win) or 15 minutes (win%).

The reason why only DTW and Xcorr lead to good results for the spectra of harpy eagle vocalizations is probably that these two methods are the only ones that compare two-dimensional matrices. Therefore, the spectra did not have to be transformed into a one-dimensional vector (Figure 5). Thus, the two-dimensional pattern visible in the spectrogram is preserved. This could also be the reason why the spectrum alone, which was used via the All-in-one procedure, did not lead to sufficient results.

### 4.1. All-in-One vs. Windowed

If the All-in-one procedure is to be used, the results show that it works best in combination with fuzzy clustering and extracted acoustic features. The fact that fuzzy clustering can best handle All-in-one data can probably be explained by fuzzy cluster overlap, which enables the algorithm to compare the degree of gradation within and between vocalizations [25]. Thus, even slightly frequency-modulated vocalizations could be classified using All-in-one-extracted acoustic features. For all other clustering methods, at least one of the windowed procedures (win or win%) performed better, especially concerning frequency-modulated vocalizations, such as those of the giant otters; therefore it seems to be more appropriate to cluster audio signals in a windowed manner. Taking into account both the quality of the label (NMI values) and the estimated number of clusters (Δ-Cluster), the windowed procedures allowed for better clustering of the vocalizations in our study (Figure 6; Table 2 and Table 3). 

How the data transformation should be windowed in general (win or win%) cannot be reliably determined from the results. Since different numbers of windows do not matter with DTW and Xcorr, because matrices of different sizes can also be compared with each other, one could assume that, in general, windowing with windows of the same size and thus matrices of different sizes (win) would be more useful. According to the spectrogram, this statement seems to be true. However, the results show that win% can also lead to better results with these two methods when using extracted acoustic features. This could be because the positions of the windows were different, especially for vocalizations whose acoustic feature values change strongly throughout the vocalization, as with the cohesion calls, resulting in different acoustic feature values. On the other hand, it is known that DTW affords particular weight to the duration of a signal [57]. However, this weighting can only come into effect with different numbers of windows (win), and would be irrelevant with an equal number of windows (win%). The different number of windows per vocalization can be problematic with other methods (kNN, HDBSCAN, and fuzzy-clustering), especially for vocalizations that differ greatly in their durations, as the number of windows must be adjusted to the longest vocalization. Since this is achieved by inserting zeros, vocalizations that differ in their characteristics, but are both of relatively short durations, may be classified as similar due to the constant repetition of zeros. For this reason, when clustering vocalizations of different lengths, it is recommended to vary the window size between vocalizations, but also to choose the same number of windows for each vocalization (win%). However, if the vocalizations are all of similar duration, as was the case for the cohesion calls of the giant otters, it seems advisable to choose a uniform window size (win).

For acoustic features extracted in a windowed manner, kNN provided very good results in combination with CD and AP. Together with DTW and Xcorr, these methods were able to reliably cluster both the harpy’s vocalizations and the cohesion calls of the giant otters. HDBSCAN, on the other hand, only achieved good performance in clustering the vocalizations of harpies. Using All-in-one, HDBSCAN even achieved an NMI value of 1, i.e., all vocalizations were correctly assigned. However, this only applied to three out of the five clusters found. All other vocalizations were regarded as outliers and rejected, which made the result unusable for determining a repertoire in which most vocal types should be found. For windowed acoustic features, the NMI dropped to 0.7735, but this was still a high value and almost all clusters were found (Δ-Cluster = 1). It is quite possible that, with a larger amount of data, HDBSCAN would be able to determine a sufficient number of usable vocalizations for each vocal type, even with All-in-one-extracted acoustic features, which would lead to a better result. A too-small data set could also be the reason why HDBSCAN was not able to cluster the cohesion calls of the giant otters sufficiently. With a large number of vocalizations, discarding outlier vocalizations is very likely to be an advantage; however, if having the correct number of clusters is important, discarding could be a disadvantage in case there are not enough data.

### 4.2. Dimensions Reduction

A reduction in the number of dimensions for acoustic features, for example, by using principal component analysis (PCA), was deliberately omitted in this study since such a reduction could lead to worse results when clustering vocalizations [25,34]. In general, dimension reduction is only recommended if one is aware of the weaknesses of the algorithm [34]. Nevertheless, it is not advisable to use as many acoustic features as possible because redundant acoustic features or acoustic features that are not problem-related can worsen the clustering result [30]. Since windowing and subsequent transformation results in a large number of dimensions from a manageable number of acoustic features, the features can be well-chosen, but the number of dimensions can bring a corresponding algorithm to its limits. Some sources mention an upper limit of 100 dimensions as a recommendation for HDBSCAN [44]. In our case the windowed data had up to 2418 dimensions; therefore, we tested if a reduction to 100 dimensions led to a better result. For dimension reduction, we used a state-of-the-art graph-based manifold learning algorithm called t-SNE [58], for which a successful dimensional reduction for clustering vocalizations using HDBSCAN had already been demonstrated by Sainburg et al. 2020 [44]. However, the results achieved with this method were considerably worse (win%: NMI = 0.16, Δ-Cluster = 3), which is why we could not confirm a better performance by dimension reduction using graph-based manifold learning algorithms. However, a reduction in the number of windows used for feature extraction to only 4 (7 with overlap, corresponding to 189 dimensions) yielded significantly better results. In this case, the NMI could be improved from 0.77 to 1.0 using HDBSCAN with a Δ-Cluster = 1. A further reduction to 135 dimensions (3 windows, 5 with overlap) resulted in an NMI of 0.9646 with a Δ-Cluster of 1. Unfortunately, this increase in performance could only be achieved with the vocalizations of the harpies, while applying the reduction in the number of windows to the cohesion calls of the giant otters did not result in any improvement. Since the value of acoustic features of cohesion calls varied greatly throughout the vocalization, a resolution of four (respectively seven) windows is probably not sufficient, and a method that can cope better with high-dimensional data is recommended.

Problems with high-dimensional data are also known for fuzzy clustering [59]. This could explain why fuzzy clustering could not achieve good results when the data were windowed. Therefore, we reduced the dimensions of the windowed data once again to 100 using t-SNE and further reduced the number of windows to 4 (respectively 7) for feature extraction. In both cases, however, no better result could be achieved (t-SNE(win%): NMI = 0.1, Δ-Cluster = 2; 4-windows: NMI = 0.71, Δ-Cluster = 3). Winkler et al. suggested adjusting the value of the fuzzy parameter µ for high-dimensional data using the following equation:

(7) $$\mu =\;1+\frac{2}{D}$$

where *D* is the number of dimensions [59]. However, this did not lead to a better result either.

To better distinguish and classify very similar vocalizations, such as individually distinguishable vocalizations of the same vocal type, future studies could improve the clustering results by reducing the number of dimensions using sparse coding. Sparse coding can, for example, be implemented using the lasso algorithm and makes it possible to specifically identify the acoustic features of the vocalizations based on which the vocal types can be reliably distinguished from one another [27,60]. This offers the advantage of reducing the number of dimensions and could improve the classification, as specific acoustic features that should be used to differentiate the vocalizations gain in weight, and non-expedient acoustic features can be discarded.

### 4.3. Reliability of Resulting Cluster Solutions

A general problem with automated clustering methods is a lack of precise knowledge of how reliable the result is. One way to calculate the quality of the cluster solution is to determine the silhouette value. If this value is relatively high, a high-quality result could be assumed. However, relying solely on the silhouette value is not recommended, as studies have already shown [25]. The evaluations carried out here sometimes showed large deviations between the silhouette value of a cluster solution and the actual quality (measured using the NMI and Δ-Cluster values) as well. Here, we show a better approach to verifying the validity of a cluster solution by clustering the data repeatedly using different algorithms each time. The comparison of clustering results using NMI showed that, for both harpy eagle and giant otter vocalizations, a proper number of clusters and valid labeling of vocalizations can be determined, even when no a priori classification is available. Particularly in the case of challenging vocalizations, misleading results due to the selection of an unsuitable clustering method can be avoided in this manner.

### 4.4. Limitations and Future Opportunities

It should be mentioned that vocalizations signaling the identity of the individual could be graded, and thus their acoustic features could vary depending on context or related to arousal [61,62,63]. If the differences between the individually distinguishable vocalizations are only marginal, gradations of this kind could have the effect that the clustering algorithm distinguishes vocalizations from the same individual anyway, resulting in too many clusters.

Similarly, it should be noted that the determination of a vocal repertoire should not be based on acoustic features alone. Thus, the definition of a unit is also dependent on perceptual mechanisms in addition to production mechanisms [17]. For example, the meaning for the receiver of a vocalization can be determined by playback experiments [46]. In this way, vocal types can be assigned to specific contexts.

Errors may occur during the calculation of some acoustic features, as a result of which the determined feature values no longer correspond to the real acoustic parameters. This can occur, for example, when determining the fundamental frequency or the frequency bands. If the corresponding frequencies are only weakly pronounced or masked by noise, the calculation becomes more difficult. Additionally, with vocalizations that are not or only conditionally harmonic, a calculation of these acoustic features is difficult or even not useful at all. Therefore, it is often advisable to check the determined acoustic features manually. For this purpose, the software CASE outputs various graphics in which the courses of certain determined features, such as the frequency bands, are displayed.

The spectrogram was used here to represent the time–frequency. In some studies, however, the use of a cochleagram has proven to be advantageous [64]. The cochleagram uses a gammatone filter bank based on the frequency selectivity of the cochlea of humans to match the time–frequency representation to human perception. However, frequency selectivity occurs differently in vertebrates [65], and differs even among primates [66]. To assess the vocalizations neutrally, a spectrogram without any filter banks was used in this study. Nevertheless, using the software CASE, it is possible to choose whether to use a cochleagram, a mel spectrogram, or a spectrogram without an additional filter bank. Accordingly, the vocalizations of the harpy eagles and the giant otters were experimentally clustered using cochleagrams. For the harpy eagles, however, there was hardly any improvement when compared with the cluster solutions with spectrograms (NMI + 0.009 points on average). For giant otters, only clustering using kNN + CD showed a considerable improvement using the cochleagram (NMI + 0.084 points). However, better results were still achieved by clustering using windowed acoustic features. The slight improvement compared with the spectrogram possibly resulted from the fact that giant otters are mammals and thus have perceptions closer to humans than that of birds.

Many of the currently developed software solutions use trained, supervised classification procedures to detect known vocalizations [67,68]. However, these cannot be used in studies where pre-labeled vocalizations are not available, such as when creating a vocal repertoire, estimating biodiversity in an area where the entirety of vocalizations is unknown, or estimating population size using individually distinguishable vocalizations. In addition, even newer software solutions use one-dimensional vectors or just the spectrogram for classification [67,69]. The windowed and multidimensional way of extracting acoustic features presented here may not only improve the classification of vocalizations, but also has advantages in other areas for future studies. Since vocalizations can be described in more detail as a result, changes within a vocal unit can be better detected. This would allow for the better definition of units, not only by silent gaps, but also by changes in frequency bands, for example. This offers the possibility to divide vocalizations more precisely to possibly find several meanings within a vocalization separated by silent gaps, or to detect sequences even in linked units. The computer-aided subdivision of units has the additional advantage of making acoustic signal analysis more efficient, standardized, and reproducible [34].

The data sets used here were comparatively small. More vocalizations from more animals are needed to determine a vocal repertoire, and the number of clusters can also be considerably larger. More clusters can also be expected when estimating population sizes in the wild. Some clustering algorithms are known to become less accurate as the number of clusters increases [70]. In general, big data pose a problem for clustering algorithms. According to Bezdek and Hathaway, the size of big data starts at around 100 Gb [71,72]. Taking the vocalizations used here as a basis, even the largest multidimensional data matrix (obtained using spectrograms), which serves as an input for the clustering algorithms, requires an average of 0.34 MB per vocalization. To reach the big data level, more than 290,000 vocalizations would have to be clustered. If acoustic features are extracted, the size of the feature matrix drops to 0.006 MB per vocalization. Accordingly, over 1.5 million vocalizations would push the algorithms to their limits. Even close to large numbers of vocalizations are probably rarely achieved. We applied the software CASE to a dataset of 757 grunts from 27 male baboons. The grunts of male baboons are known to differ individually. The software CASE was able to identify 25 clusters using HDBSCAN. However, since these vocalizations were not frequency-modulated and more data sets would have become confusing for the reader when comparing the algorithms, this data set was not considered useful.

The main goal of this study was to provide future conservation projects, but also basic researchers, with the possibility to obtain results non-invasively and as accurately and reliably as possible. The freely available software CASE offers easy use of complex algorithms and a variety of acoustic features for the detailed characterization of animal sounds. The variety of methods included should allow for addressing specific questions and challenges as flexibly as possible. In this way, we want to encourage researchers to contribute to the understanding of animal communication and conservation using bioacoustic studies.

## 5. Conclusions

Based on the findings of this study, it can be recommended that, especially with frequency-modulated vocalizations, audio data should be windowed to achieve better results. If windowing is not possible or not desired, fuzzy clustering (and with a sufficient amount of data, probably also HDBSCAN) can also achieve good results with All-in-one-transformed audio data if only slightly frequency-modulated vocalizations are used, but acoustic features should then be extracted, rather than using the spectrum. In general, however, windowing is also recommended for slightly frequency-modulated vocalizations. In this case, both extracted acoustic features and time–frequency spectra can lead to good results. When using spectra, dynamic time warping or cross-correlation in combination with community detection or affinity propagation should be used. For extracted acoustic features, the best results were achieved by k-nearest neighbor and dynamic time warping in combination with community detection or affinity propagation, but also by HDBSCAN. If HDBSCAN is used, however, care should be taken to keep the number of windows as small as possible to achieve no more than 200 dimensions.

If vocalizations are highly frequency-modulated, the windowing should have as high a resolution as possible, and classifiers should be used that can process high-dimensional data sets better. Here, k-nearest neighbor in combination with community detection or affinity propagation, but also dynamic time warping in combination with community detection, achieved good results. Whether the audio signals are all processed with the same window size and, accordingly, with a different number of windows (win), or whether the number of windows should be the same for all vocalizations (win%) should be decided depending on the duration of the different vocalizations, but should also be chosen depending on the classifier and the clustering method used. In general, and especially if vocal types vary in duration, the same number of windows should be chosen for all vocalizations (win%). However, in some cases, if all vocalizations have approximately the same length and differ only slightly from one another, it can be advantageous to fix the window size and leave the number of windows per vocalization variable (win). Since DTW and Xcorr can compare matrices with a different number of windows, and thus no zeros have to be appended to “win” prepared data, these two procedures are the only ones where no disadvantages arise due to appended zeros, even with vocalizations of different lengths. 

To ensure that the clustering result is robust, we recommend that several runs with different algorithms are applied to the same data set, followed by a comparison of the results. To compare the labels, NMI can be used. If the NMI value is high, the clustering result is substantiated. For a quick and easy application of the procedures described here, the software CASE is available for download. This tool contains all clustering methods, as well as the data transformation procedures (All-in-one, win, and win%) and feature-extraction algorithms. Thus, the advantages of windowing audio data and the comparison of several cluster solutions of different methods can be used by a wide range of research groups to obtain more robust results. The download link to the freely available software CASE can be found in the supporting information.

## Figures and Tables

**Table 1:** List of acoustic features extracted for one vocalization (All-in-one) or for each time window of one vocalization (windowed). The commands (in quotes) and toolboxes mentioned in the reference column all refer to MATLAB.

Acoustic Features	Definition of Features (All-in-One)	Definition of Features (Windowed)	Reference
F0	Median fundamental frequency	Fundamental frequency for each window	Audio Toolbox “pitch” with NCF
Delta F0	Median value of the difference between adjacent values for F0 per window	Value of the difference between adjacent values for F0	
Dominant f	Frequency with highest amplitude in the spectrum	Frequency with highest amplitude in the spectrum of each window	
Min f	Lower bound of the 99% occupied bandwidth	Lower bound of the 99% occupied bandwidth	Signal Processing Toolbox “obw”
Max f	Upper bound of the 99% occupied bandwidth	Upper bound of the 99% occupied bandwidth	Signal Processing Toolbox “obw”
Bandwidth	99% occupied bandwidth	99% occupied bandwidth	Signal Processing Toolbox “obw”
Duration	Time between onset and offset of a vocalization in seconds.	Duration only determined for win%, not for win.	
1st Quartile	1st quartile of the energy distribution	1st quartile of the energy distribution	[47]
2nd Quartile	2nd quartile of the energy distribution	2nd quartile of the energy distribution	[47]
3rd Quartile	3rd quartile of the energy distribution	3rd quartile of the energy distribution	[47]
Max Q1	Frequency with highest amplitude in the 1st quartile	Frequency with highest amplitude in the 1st quartile	
Max Q2	Frequency with highest amplitude in the 2nd quartile	Frequency with highest amplitude in the 2nd quartile	
Max Q3	Frequency with highest amplitude in the 3rd quartile	Frequency with highest amplitude in the 3rd quartile	
Max Q4	Frequency with highest amplitude in the 4th quartile	Frequency with highest amplitude in the 4th quartile	
FB1	Median frequency of the 1st frequency band determined by LPC (Hz)	Frequency of the 1st frequency band determined by LPC	Signal Processing Toolbox “lpc” with 205 coefficients
FB2	Median frequency of the 2nd frequency band	Frequency of the 2nd frequency band	LPC with 205 coefficients
FB3	Median frequency of the 3rd frequency band	Frequency of the 3rd frequency band	LPC with 205 coefficients
BW FB1	Median bandwidth of FB1	Bandwidth of FB1	“findpeaks”
BW FB2	Median bandwidth of FB2	Bandwidth of FB2	“findpeaks”
BW FB3	Median bandwidth of FB3	Bandwidth of FB3	“findpeaks”
Delta FB1-FB2	Difference between FB1 and FB2	Difference between FB1 and FB2	
Delta FB2-FB3	Difference between FB2 and FB3	Difference between FB2 and FB3	
Number of FB	Number of frequency bands determined by LPC	Number of frequency bands determined by LPC	Number calculated by LPC
Harmonic Ratio	Harmonic ratio is returned with values in the range of 0 to 1. A value of 0 represents low harmonicity, and a value of 1 represents high harmonicity	Harmonic ratio is returned with values in the range of 0 to 1. A value of 0 represents low harmonicity, and a value of 1 represents high harmonicity	Audio Toolbox “harmonicRatio”
Spectral Flatness	Measures how noisy a signal is. The higher the value, the noisier the signal	Measures how noisy a signal is. The higher the value, the noisier the signal	Audio Toolbox “spectralFlatness”
MFCC 1	1st mel frequency cepstral coefficient	1st mel frequency cepstral coefficient	Audio Toolbox “mfcc”
MFCC 2	2nd mel frequency cepstral coefficient	2nd mel frequency cepstral coefficient	Audio Toolbox “mfcc”

**Table 2:** NMI values of the comparison between the labeling of the different clustering methods and the actual allocation of vocalizations to the corresponding giant otter individuals. The allocation was not human-rated, but could be determined by sound localization. Only those NMI values that were achieved by clustering methods that met the threshold values for both NMI and Δ-Cluster are shown.

Clustering Methods	Acoustic Features			Spectra			Data Set
	Win	Win(%)	All-in-One	Win	Win(%)	All-in-One	
**kNN + CD**	0.80	0.61					Giant Otter
**kNN + AP**	0.61						
**DTW + CD**	0.50						
**DTW + AP**	0.50						
**Xcorr + CD**							
**Xcorr + AP**	0.58						
**HDBSCAN**							
**fuzzy**							

**Table 3:** NMI values of the comparison between the labeling of the different clustering methods and the authors’ rated label. Only the NMI values that were achieved by clustering methods that met the threshold values for both NMI and Δ-Cluster are shown.

Clustering Methods	Acoustic Features			Spectra			Data Set
	Win	Win(%)	All-in-One	Win	Win(%)	All-in-One	
**kNN + CD**	0.67	0.92					Harpy Eagle
**kNN + AP**		0.85	0.79	0.51			
**DTW + CD**		0.78	0.67	0.75	0.54		
**DTW + AP**	0.65		0.61	0.64	0.59		
**Xcorr + CD**		0.78		0.79			
**Xcorr + AP**	0.65			0.74	0.87		
**HDBSCAN**		0.77					
**fuzzy**			0.85				

**Table 4:** Comparison of the determined harpy eagle vocalization labels using NMI. NMI values are shaded if NMI > 0.5 and Δ Cluster ≤ 1.

	Acoustic Features (Win%)					Spectrogram (Win%)		Acoustic Features (All-in-One)
	kNN + CD	kNN + AP	DTW + CD	DTW + AP	HDBSCAN	Xcorr + CD	Xcorr + AP	Fuzzy
**kNN + AP**	0.90							
**DTW + CD**	0.70	0.65						
**DTW + AP**	0.64	0.63	0.86					
**HDBSCAN**	0.68	0.68	0.92	0.88				
**Xcorr + CD**	0.91	0.81	0.65	0.60	0.68			
**Xcorr + AP**	0.88	0.79	0.68	0.62	0.64	0.84		
**Fuzzy**	0.78	0.72	0.73	0.67	0.76	0.80	0.74	

**Table 5:** Comparison of the determined giant otter vocalization labels using NMI. NMI values are shaded if NMI > 0.5 and Δ Cluster ≤ 1.

	Acoustic Features (Win%)					Spectrogram (Win%)		Acoustic Features (All-in-One)
	kNN + CD	kNN + AP	DTW + CD	DTW + AP	HDBSCAN	Xcorr + CD	Xcorr + AP	Fuzzy
**kNN + AP**	0.64							
**DTW + CD**	0.56	0.45						
**DTW + AP**	0.56	0.45	1					
**HDBSCAN**	0.4	0.42	0.10	0.10				
**Xcorr + CD**	0.37	0.46	0.41	0.41	0.46			
**Xcorr + AP**	0.41	0.43	0.41	0.41	0.32	0.67		
**Fuzzy**	0.57	0.59	0.51	0.51	0.47	0.63	0.42	

**Table 6:** NMI and Δ-Cluster values achieved by inexperienced, human observers compared with the a priori labels.

	Harpy Eagle		Giant Otter	
Person	NMI	Δ-Cluster	NMI	Δ-Cluster
**A**	0.9233	1	0.3013	1
**B**	0.9293	0	0.5079	3
**C**	0.8527	1	0.381	1
**D**	0.9545	0	0.3236	0
**E**	0.9233	1	0.3877	0

## Data Availability

The audio recordings presented in this study are available on request from the corresponding author. The data are not publicly available, since the audio recordings may contain voices of zoo visitors or zoo staff whose consent for publication we do not have.

## Appendix A

Download link for the software CASE: https://de.mathworks.com/matlabcentral/fileexchange/88039-case-cluster-analyse-sound-events.

The extracted acoustic features can be accessed at the following DOI: https://doi.org/10.6084/m9.figshare.20359083.v1.
